# An mHealth App for Supporting Quitters to Manage Cigarette Cravings With Short Bouts of Physical Activity: A Randomized Pilot Feasibility and Acceptability Study

**DOI:** 10.2196/mhealth.6252

**Published:** 2017-05-26

**Authors:** Mary Hassandra, Taru Lintunen, Martin S Hagger, Risto Heikkinen, Mauno Vanhala, Tarja Kettunen

**Affiliations:** ^1^ Faculty of Sport and Health Sciences University of Jyvaskyla Jyvaskyla Finland; ^2^ School of Psychology and Speech Pathology, Faculty of Health Sciences Health Psychology & Behavioral Medicine Research Group & Laboratory of Self-Regulation (Laser) Curtin University Perth Australia; ^3^ Department of Physical Education Hong Kong Baptist University Hong Kong China (Hong Kong); ^4^ Statistical Analysis Services Analyysitoimisto Statisti Oy Jyvaskyla Finland; ^5^ School of Medicine Unit of Primary Health Care University of Eastern Finland Kuopio Finland; ^6^ Unit of Primary Health Care Central Finland Health Care District Jyvaskyla Finland; ^7^ Faculty of Sport and Health Sciences Health Sciences University of Jyvaskyla Jyvaskyla Finland

**Keywords:** behavior change, mHealth app, physical activity, randomized controlled trial, relapse prevention, smoking

## Abstract

**Background:**

While gains in reducing smoking rates in Finland have been made, prevalence rates are still substantial. Relapse rates among smokers engaged in quit-smoking programs are high. Physical activity has been proposed as one means to help smokers manage cravings. Software and apps on mobile phone and handheld devices offer an opportunity to communicate messages on how to use physical activity to manage cravings as part of quit-smoking programs.

**Objective:**

We aimed to test the feasibility, acceptability, usability, and preliminary efficacy of an mHealth mobile phone app, Physical activity over Smoking (PhoS), to assist smokers in quitting smoking in a randomized controlled trial. The app was designed to prompt smokers to engage in physical activities to manage their smoking cravings.

**Methods:**

Regular smokers (n=44) attended a group-based behavioral counselling program aimed at promoting physical activity as an additional aid to quit. After quit day, participants were randomly allocated to an intervention (n=25) or to a comparison (n=19) group. Participants in the intervention group were provided with the PhoS app and training on how to use it to assist with relapse prevention. Participants in the comparison condition were provided with generalized relapse prevention training.

**Results:**

Some participants reported that the PhoS app was useful in assisting them to successfully manage their cigarette cravings, although compliance across the sample was modest and participants reported low levels of usability. Participants receiving the PhoS app did not report greater abstinence than those who did not receive the app. However, participants receiving the app were more likely to report greater abstinence if they did not use pharmacological support, while those who did not receive the app reported greater abstinence when using pharmacological support. Participants receiving the app reported greater levels of physical activity than those who did not. Results revealed that the app resulted in better retention.

**Conclusions:**

The PhoS app showed some potential to reduce abstinence among participants not using pharmacological therapy and to increase physical activity. However, problems with usability and lack of effects on abstinence raise questions over the app’s long-term effectiveness. Future research should prioritize further development of the app to maximize usability and test effects of the intervention independent of quit-smoking programs.

**Trial Registration:**

International Standard Randomized Controlled Trial Number (ISRCTN): 55259451; http://www.controlled-trials.com/ISRCTN55259451 (Archived by WebCite at http://www.webcitation.org/6cKF2mzEI)

## Introduction

The harmful effects of smoking on health are well documented, but quit rates are low and long-term relapse rates range from 75%, for smokers adopting combined therapies of counseling and pharmacotherapy to assist quitting, to 95%, for smokers who adopt a complete abstinence (“cold turkey”) strategy without pharmacological or therapeutic support [1-3]. A systematic review of the effectiveness of smoking relapse prevention interventions [4] indicated that self-help treatments can aid relapse prevention. Mobile phone apps have been used either as a stand-alone treatment program to assist quitting or as a self-help tool in combination with other treatment programs [5]. Behavioral interventions delivered through mobile phones and handheld devices offer promising opportunities to expand psychological practice [6,7], and the integration of effective behavior change interventions using software in these devices [8] (collectively known as mHealth apps) may help develop stronger evidence in future research in this field.

Physical activity has recently been incorporated into existing smoking cessation counselling programs as a cessation aid [9-12]. There is good evidence for the acute, short-term effects of physical activity on smoking-related variables. Research has indicated that physical activity acutely reduces cigarette craving [13-15]. The literature suggests a wide range of intensities, from isometric exercises and yoga to high-intensity activities with heart rates of up to 85% of maximum, as an aid to managing cigarette cravings and withdrawal symptoms [13-17]. However, although evidence of the positive effects of physical activity on reducing cravings is promising [13-16], most of the research focuses on acute, short-term effects, and findings are limited by a lack of long-term follow-up and a focus on laboratory-based studies. Therefore, there is a need for interventions to examine the long-term effects of physical activity on cigarette cravings and smoking cessation in real-life situations. Similar efforts in this line of research have been initiated [17,18], but none have adopted mobile technology and mHealth apps.

The use of mHealth apps may provide a valuable platform to test the feasibility of physical activity interventions to reduce smoking cravings and promote quitting in real-life contexts. To serve this purpose, we developed an mHealth app, Physical activity over Smoking (PhoS), to support smokers trying to quit in managing their cigarette cravings and abstaining in the long term using short bouts of physical activity as a behavioral substitution strategy. We based the PhoS app on psychological theory and evidence-based relapse prevention strategies [19]. Following its development, we aimed to assess the feasibility and acceptability of the PhoS app for use by quitting smokers, and to test its effects in changing outcome measures (abstinence, self-reported cravings, relapses, and awareness of cravings) in a small-scale randomized controlled trial (ISRCTN55259451; Multimedia Appendix 1 [20]).

The aim of this preliminary study was to assess the feasibility, acceptability, and preliminary efficacy of the newly developed mHealth app PhoS. We predicted that use of the app would be feasible as a means to assist smokers wanting to quit and would have high levels of acceptability consistent with research using mHealth apps in other behavioral health contexts. We also hypothesized that smokers using the app would have high abstinence rates, lower self-reported cravings and relapses, and greater awareness of cravings.

## Methods

### Participants, Procedure, and Randomization

We screened participants for eligibility by having them complete a short battery of questions online. Eligibility criteria were age (adults 18-65 years), regular smoking (smoking >10 cigarettes/day), no existing mental health condition or other addictions, expression of high motivation to quit, and no health conditions for which physical activity was contraindicated. Noneligible participants were referred to their general practitioner for further health advice. We invited participants who met the eligibility criteria and gave verbal consent to participate, via a telephone call, to take part in a smoking cessation program, consisting of 3 weekly counseling sessions, and helped them set a quit date. Participants who reached the quit day were randomly allocated to an intervention or a comparison group. Within 3 to 7 days after their quit day, participants in both groups had a fourth session with the study nurse and were provided with group training on relapse prevention and prompted to form action plans to cope with their cravings. Participants in the intervention group received additional training for using the mHealth app. After they downloaded the PhoS app to their mobile phones, they were provided with instructions on how to use it as an additional support tool whenever they experienced cravings. Participants without a mobile phone or with one that was incompatible with the app were provided with a mobile phone with the PhoS app preloaded for the duration of the study. Those allocated to the comparison group were provided with guidance to develop an action plan to overcome relapses. The intervention group received the same training but with an emphasis on identifying short bouts of physical activity that can be used in everyday-life situations to overcome cravings. Then, they were provided with instruction on the use of the PhoS app as a support tool.

We randomly allocated participants to the intervention or the comparison group using version 4 of Research Randomizer, an online randomization tool [21]. The principal investigator generated the allocation sequence and the study nurse assigned participants to groups. The principal investigator was blinded to the participants’ data up to the first follow-up occasion, and the study nurse was blinded to the allocation sequence until the day of randomization. Participants were blinded to group assignment. Sessions took place at the Jyvaskyla Community Primary Health Care Center in Central Finland and the University of Jyvaskyla campus.

Screening data were collected via an online questionnaire. Those who were eligible received a telephone call from the study nurse to set up a time for the first session at the health center or university campus to complete baseline measures after they agreed to participate by signing the consent form. We collected data from the face-to-face sessions via a paper-and-pencil questionnaire. Follow-up data were collected via online questionnaires after an invitation from the research assistant via email or text message, which contained a link to the online questionnaire. App users’ data were automatically uploaded daily to a secure university server reserved for this purpose. We obtained ethics approval from the Ethics Committee of the Central Finland Health Care District (Keski-Suomen Sairaanhoitopiirin Eettinen Toimikunta).

### Intervention

The intervention has been described in detail in the published study protocol [19]. The main theoretical frameworks used for the intervention and app development were sociocognitive theory [22], the theory of planned behavior [23], control theory [24], the relapse prevention model [25], and motivational interviewing [26]. The behavior change techniques based on these theoretical approaches that we used in the interventions were self-monitoring, setting goals, setting graded tasks, reviewing behavioral goals, and planning action and coping.

The majority of the sessions were group based, but we organized some individual sessions for those who missed the group sessions. All sessions were delivered by the study nurse, who had previous experience with counseling smokers to quit and an additional 3 hours of training from the research group on how to deliver the specific intervention. In most of the sessions, a member of the research group who was also an accredited psychologist was also present and kept notes throughout the procedure to ensure adherence to the protocol and participated in group discussions related to the promotion of physical activity if needed.

The first 3 sessions, common to both groups, aimed to help participants quit smoking. The promotion of physical activity as a supportive behavior to decrease, and eventually quit, smoking was a central part of these sessions. The aim of the fourth session, which was held after random allocation to the intervention and comparison groups, was to support participants in developing relapse prevention plans and action plans to manage cravings. The only difference between the 2 groups in this fourth session was that, for intervention group there, we emphasized using short bouts of physical activity (taking a brisk walk, stretching, breathing, balance, etc) as the main craving management strategy.

The PhoS app was introduced as a tool that would give them ideas of what physical activities to do and how to do them depending on their individual and situational conditions. The app drew from a database that contained strategies based on the relapse prevention model [27,28] and the taxonomy of behavior change techniques for smoking cessation [29]. The app database includes a pool of 57 introductory messages (eg, “Researchers have found 70 poisonous chemicals in cigarettes which cause cancer. Stay healthy!”), 49 motivational messages (eg, “feel like a winner, not miserable after 3 minutes!”), and 64 physical activities (eg, “Stretch your upper arms. Hold for 10 seconds each”), all of which were coded to appear according to the users’ profile and status.

Data entries were self-initiated whenever participants used the app. All usage data were automatically uploaded to the server whenever the device was connected to the Internet. Participants’ identification number and profile settings were entered when the PhoS app was installed on their mobile phones and used for the first time. After that, every time users reported experiencing a craving, they were prompted to provide situational information regarding mood, place, and social environment. According to users’ profile information and the situational status, a variety of physical activities accompanied by theory-based motivational messages were suggested. Physical activities were animated to demonstrate the suggested activities in a visual form. Finally, each time participants used the app, they were asked to provide feedback if they had successfully managed the craving. Screenshot 1 in Multimedia Appendix 2 provides details of the profile settings (eg, sex, age, days since quit date) screen. Screenshot 2 provides an example of the situational data users could enter each time they used the app (mood, place, and social environment). Screenshot 3 displays some examples of the animated physical activities provided to participants to demonstrate the activities. Finally, screenshot 4 provides an example of the feedback users were given after they had used the app.

Participants’ use of the app was monitored by the PhoS app, which automatically uploaded participants’ data to a central server. When participants had not used the PhoS app for 1 week, they were contacted to check whether there was a technical problem or they had stopped using the app. If they reported that they were not using the app any longer, they answered a short questionnaire to assess the reasons for discontinuing its use.

### Measures

#### Implementation Outcomes

We assessed engagement with the PhoS app through data extracted from participants’ mobile phones indicating frequency and duration of app use. Moreover, we calculated retention rates of the app users for the same time points as the other follow-up measures.

We assessed fidelity of app use through open- and closed-ended questions to record whether participants had used the app as instructed, as well as questions regarding any other additional support they used (eg, pharmacological, other mHealth apps). Additional usage monitoring data were extracted from the phones to describe usage. Fidelity was assessed at 3 days and at 1, 3, and 6 months after participants had started using the app.

We assessed usability of the app twice, at 1 week and 1 month, after participants had started using the app. Participants completed the System Usability Scale (SUS) self-reported questionnaire [30], a 10-item scale measuring subjective components of usability. Responses were provided on 5-point scales ranging from strongly agree (1 point) to strongly disagree (5 points). Moreover, participants who stopped using the app at any point during the trial were prompted to supply reasons why they had done so using an open-ended question.

#### Preliminary Efficacy Outcomes

The primary efficacy outcome was self-reported 7-day point prevalence abstinence (PPA) at 7 days prior to each scheduled follow-up [31]. PPA measures after the quit time point and at the end of the follow-up (6 months) were verified by a saliva cotinine test [32].

Secondary outcome measures were self-reported number of relapses during the last 7 days, self-reported number of cravings during the last 7 days, and self-efficacy of being aware of experiencing cravings (AEF; “How well are you aware of your cigarette cravings?”). These 3 single-question assessments were collected at the same time points as the main outcome measure. We assessed 2 more secondary outcomes: self-efficacy in managing cravings (MCEF; “How well do you manage your cravings?”) and the power of control in managing cravings (CCM). The CCM was assessed with 6 items with different permutations for the intervention and control groups. An example item is “If I am in a situation where I celebrate with my friends...,” then the answer for the intervention group was “It will be more difficult to use [the] PhoS app to control my craving for tobacco,” and for the comparison group it was “It will be more difficult to do something to control my craving for tobacco.” Responses were given on 7-point scales ranging from totally agree (1 point) to totally disagree (7 points). Multimedia Appendix 3 displays the intervention timeline and the detailed measurement time points for each group and all measures.

We also included additional outcome measures: self-reported physical activity assessed by the International Physical Activity Questionnaire (IPAQ) [33] collected at sessions 1 and 3, and at the 6 month follow-up time points; and attitude, intention, and perceived behavioral control with respect to increasing physical activity behavior [34] at sessions 1 and 4. We assessed attitude using 6 items on 7-point semantic differential scales ranging from –3 to +3 points (eg, “To increase physical activity in the following month will be for me...” good-bad, unpleasant-pleasant, wise-silly, easy-difficult, healthy-unhealthy, important-not important). We measured intention using 3 items with responses provided on 7-point scales ranging from very likely (1 point) to very unlikely (7 points) (eg, “In the following month I intend to increase physical activity”). We measured perceived behavioral control using 4 items on 7-point scales ranging from complete control (1 point) to very little control (7 points) (eg, “How much control will you exert over exercising regularly during the next month?”).

### Statistical Analyses

We conducted all main analyses using the intention-to-treat method with participants remaining in their originally assigned groups after random allocation regardless of adherence or protocol deviation. We also performed the same analyses using complete-case analysis. We report data using descriptive statistics, including mean, standard deviation, and frequencies, and used analysis of variance to test group comparisons on baseline and demographic data. Logistic regression examined differences between the groups on the dichotomous primary outcome variable: abstinence versus nonabstinence at the 6-month follow-up time point. We tested group differences at all follow-up time points using a 3 (time points: baseline, 1 week, 6 months) × 2 (groups: intervention, control) generalized linear mixed model (GLMM) with logit link function. We identified the use of pharmacological support as an important control variable in the preliminary analyses and added it to the models as a covariate. We also used a series of GLMMs of identical design to analyze the continuous secondary efficacy measures AEF, MCEF, and CCM during the follow-up period. In addition, a series of GLMMs with Poisson link function using the same design as the previous analyses analyzed the number of cigarettes and number of cravings. For number of relapses, AEF, MCEF, and CCM, nonparametric Mann-Whitney *U* tests compared differences between the intervention and comparison groups at the 6-month time point. We used R statistical package software version 3.1.3 (R Foundation) for all analyses.

## Results

### Participant Flow

**Figure 1:**
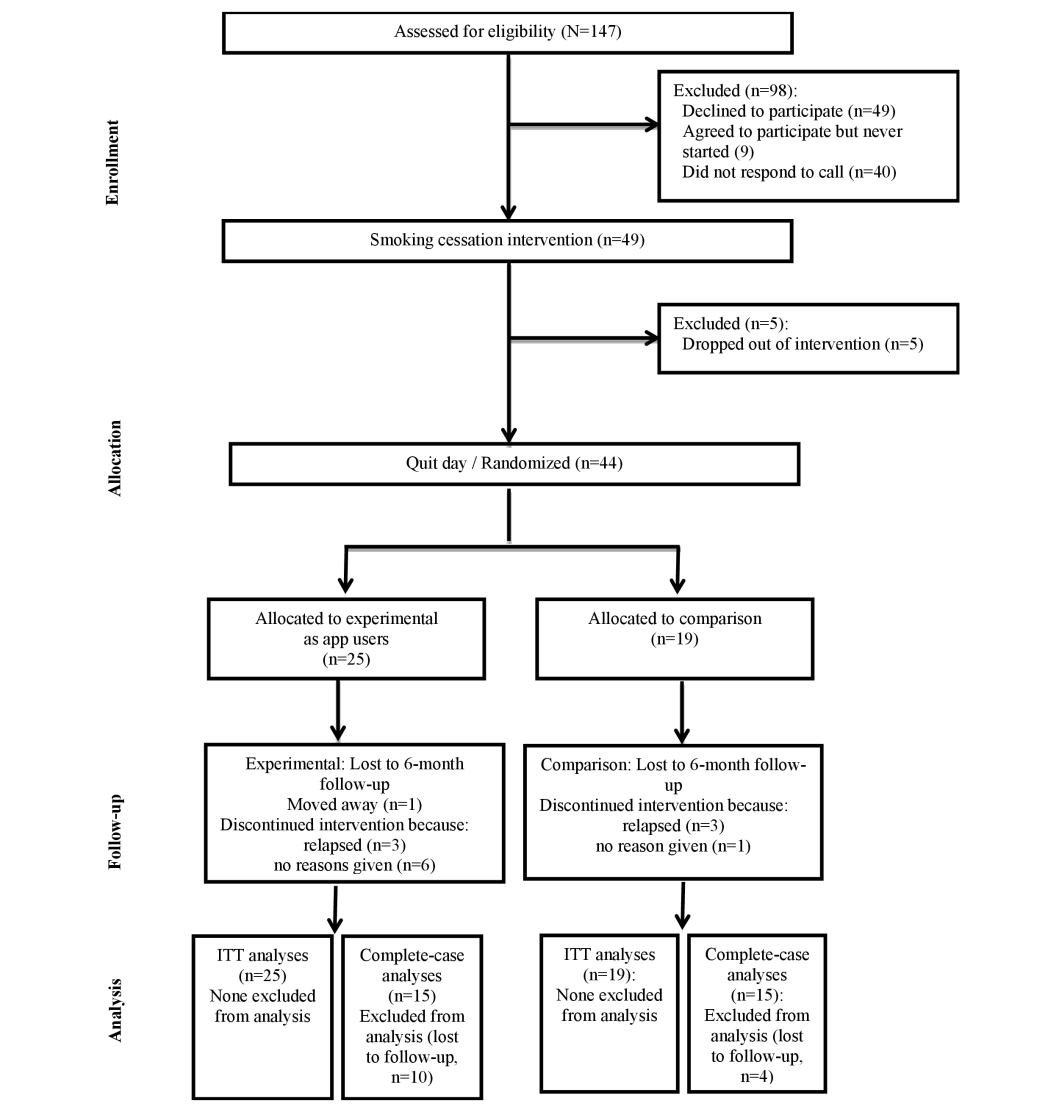
Participant flow chart. ITT: intention-to-treat.

The initial response rates in recruitment were lower than expected, so we extended the recruitment period and used several additional recruitment methods. A radio interview with the study nurse on a local radio station was the most successful and immediate recruitment strategy. Of the 147 individuals who completed the screening assessment and were eligible, 49 agreed to participate and entered the smoking cessation intervention. A total of 44 participants reported quitting and were randomly allocated into intervention (n=25) and comparison (n=19) groups. The groups were not balanced because the group allocation was an ongoing procedure and the randomization sequence was generated for 50 participants (25 for each group), but only 44 people were finally randomly allocated. Figure 1 illustrates the flow of participants. Some participants did not attend all 3 of the prequit sessions. However, we included those who attended at least one session, managed to quit, and also attended the fourth session in the follow-up group (n=4). A total of 34 participants completed all of the follow-up measures (77% retention rate); 19 of the 25 participants allocated to the intervention group completed all the follow-up measures (76% retention rate) and 15 of the 19 participants allocated to the comparison group completed all the follow-up measures (79% retention rate). The most common reason participants gave for dropping out at follow-up was relapse back to smoking and no longer wanting to continue the study.

### Baseline Data

All participants were of Finnish nationality. Six (14%) participants had a university education, 16 (36%) had an applied sciences education, 14 (31%) had a vocational school education, and 3 (7%) had a secondary school education only. Regarding employment status, 33 (75%) were employed, 3 (7%) were unemployed, 3 (7%) were pensioners, and 5 (11%) were university students. Table 1 presents additional characteristics of the study participants.

Table 2 presents descriptive measures by group for all sessions. There were no differences between groups in demographic characteristics (Table 1) or among all measures during all sessions (Table 2).

**Table 1:** Participant characteristics^a^.

Characteristics		Study group					
		All (n=44)		Intervention (n=25)		Comparison (n=19)	
		Mean or n	SD or %	Mean or n	SD or %	Mean or n	SD or %
Age in years, mean (SD)		39.11	10.67	39.92	11.16	38.05	11.16
Salary (€), mean (SD)		28,613	11,436.30	29,280	10,121.22	27,736	13,207.07
BMI^b^in kg/m^2^, mean (SD)		25.53	3.89	25.35	3.91	25.78	3.97
**Pharmacological support**							
	Used	29	66	16	64	13	68
	Not used	15	34	9	36	6	32
**Sex**							
	Male	25	57	15	60	10	53
	Female	19	43	10	40	9	47

^a^No significant between-group differences were detected in the means.

^b^BMI: body mass index.

**Table 2:** Descriptive measures (mean scores) during all sessions by group.

Measures		Study group								
		All			Intervention			Comparison		
		n	Mean	SD	n	Mean	SD	n	Mean	SD
**Session 1 (baseline)**										
	Smoking behavior 7 days^a^	41	15.35	7.30	22	14.87	8.69	19	15.90	5.44
	IPAQ^b^	41	3853.24	4719.79	22	4481.18	5527.46	19	3126.15	3580.26
	Attitude physical activity	41	1.69	.45	22	1.68	.45	19	1.71	.46
	Intention physical activity	41	1.79	.69	22	1.72	.66	19	1.86	.74
	Perceived behavioral control over physical activity	41	1.95	.64	22	1.98	.70	19	1.90	.58
	CCM^c^	41	3.23	1.24	22	3.28	1.42	19	3.17	1.04
**Session 2**										
	Smoking behavior 7 days	38	11.08	4.41	19	11.42	5.07	19	10.75	3.73
**Session 3**										
	Smoking behavior 7 days	37	8.96	4.19	19	9.35	4.27	18	8.55	4.18
	IPAQ	36	3981.66	4865.02	19	3204.10	3770.73	17	4850.70	5853.08
	CCM	37	3.60	1.34	19	3.39	1.48	18	3.82	1.17
**Session 4**										
	Smoking behavior 7 days	32	8.45	4.19	17	8.18	4.16	15	8.77	4.35
	Attitude physical activity	44	1.49	.66	25	1.54	.78	19	1.43	.47
	Intention physical activity	37	2.36	1.32	21	2.65	1.55	16	2.00	.85
	Perceived behavioral control over physical activity	37	2.25	.97	21	2.41	1.20	16	2.04	.51

^a^Data from the everyday self-monitoring diary.

^b^IPAQ: International Physical Activity Questionnaire.

^c^CCM: power of control in managing cravings.

Of the 44 participants, 38 took the saliva cotinine test, and 11 of them were verified as abstinent (index 0 or 1), based on a cutoff value of 10 ng/mL, which is the stated abstinence using NicAlert saliva cotinine tests [32]. A total of 19 of them had a medium cotinine index (2-4), and 8 had an index score of 5 or 6. All participants with a score higher than 1 stated that they were using cotinine replacements (2- to 4-mg tablets or gum 2-8 times/day) and therefore were considered as eligible to enter the follow-up period for measurements. Data on the saliva cotinine test at the 6-month follow-up were not available. We offered the participants several options to take the test (home visit, by mail, etc), but uptake was extremely low (n=5). Therefore, verification of self-reported data at the end was not possible.

### Implementation Outcomes

#### Engagement

Data from some mobile phones could not be uploaded to the server when connected to the Web, which left 14 (56%) participants’ data available for engagement and compliance analysis. We contacted participants to offer them a new phone, but they declined. We omitted phone data from 5 participants from analysis because they did not use the app at all after the initial session. We selected only fully completed cases of use of the app from the start button entry to the feedback button entry. We excluded incomplete data units, as we considered them not to be valid uses of the app. Complete phone data were extracted for 9 users. Table 3 displays the average use data at the same time points as the other follow-up measures.

**Table 3:** Average frequencies of use during the period measured at each time point.

Use	Time point							
	3 days	1 week	2 weeks	3 weeks	4 weeks	8 weeks	12 weeks	24 weeks
Duration of use (days)^a^	1.56	0.56	1.11	0.78	1.00	3.56	2.22	2.56
No. of days of use^b^	1.44	0.56	0.67	0.56	0.33	0.78	0.56	0.44
Average uses/day^c^	1.09	0.78	0.44	0.44	0.22	0.22	0.42	0.30
Total uses^d^	2.00	0.78	0.67	0.56	0.33	0.78	1.33	0.67
No. of minutes of use^e^	1.79	0.69	0.58	0.80	0.12	0.70	0.97	0.76
Views^f^	12.22	3.89	4.11	9.67	3.67	11.56	8.67	10.67

^a^Time period between the first and last day of using the app for each period of measurement.

^b^Number of different days the participant used the app.

^c^Number of times on average the user used the app in a single day.

^d^(Number of days) × (average number of uses).

^e^Number of minutes the participant used the app in total

^f^Number of different views the user has had on the screen during use.

**Figure 2:**
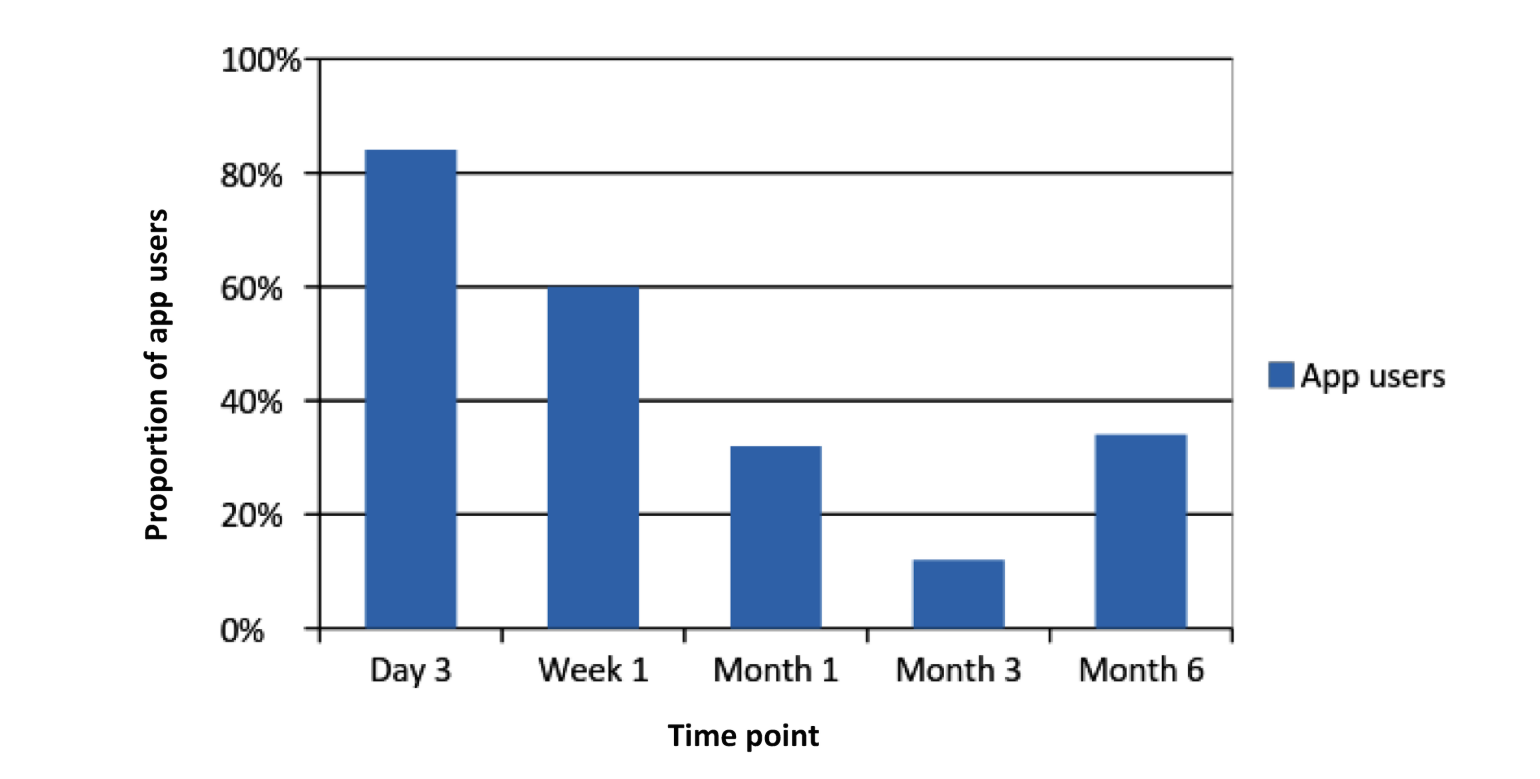
Proportion of app users during follow-up.

Figure 2 displays the retention rates for app use throughout the follow-up period. The retention rate at day 3 was the highest and at month 3 it was the lowest. We defined retention rate as the ratio of the number of retained users of the app to the number of participants who completed all study measures at the same time point.

#### Fidelity

**Figure 3:**
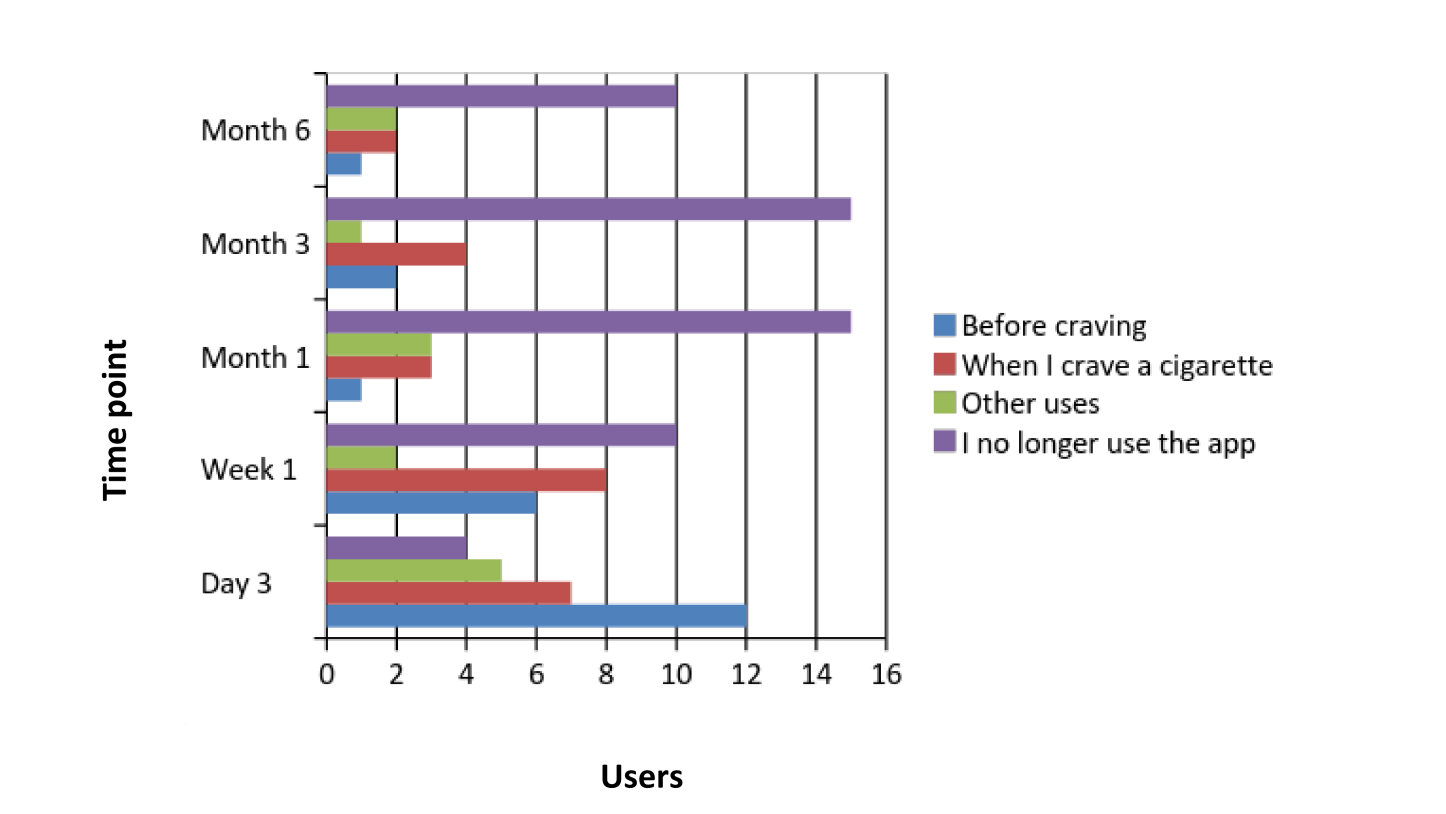
Fidelity responses of the intervention group detailing when, how, and why participants used the app.

Fidelity questions for the intervention group aimed to identify whether participants used the app, how and when they used it, and, if they decided not use it anymore, the reasons why. Figure 3 illustrates their responses to the closed-ended questions.

Participants’ (n=5) reasons for using the app were “just for fun” or “just browsing,” while reasons they stopped using it were “After the initial excitement I don’t think it is for me” or “I don’t need it any more.”

**Figure 4:**
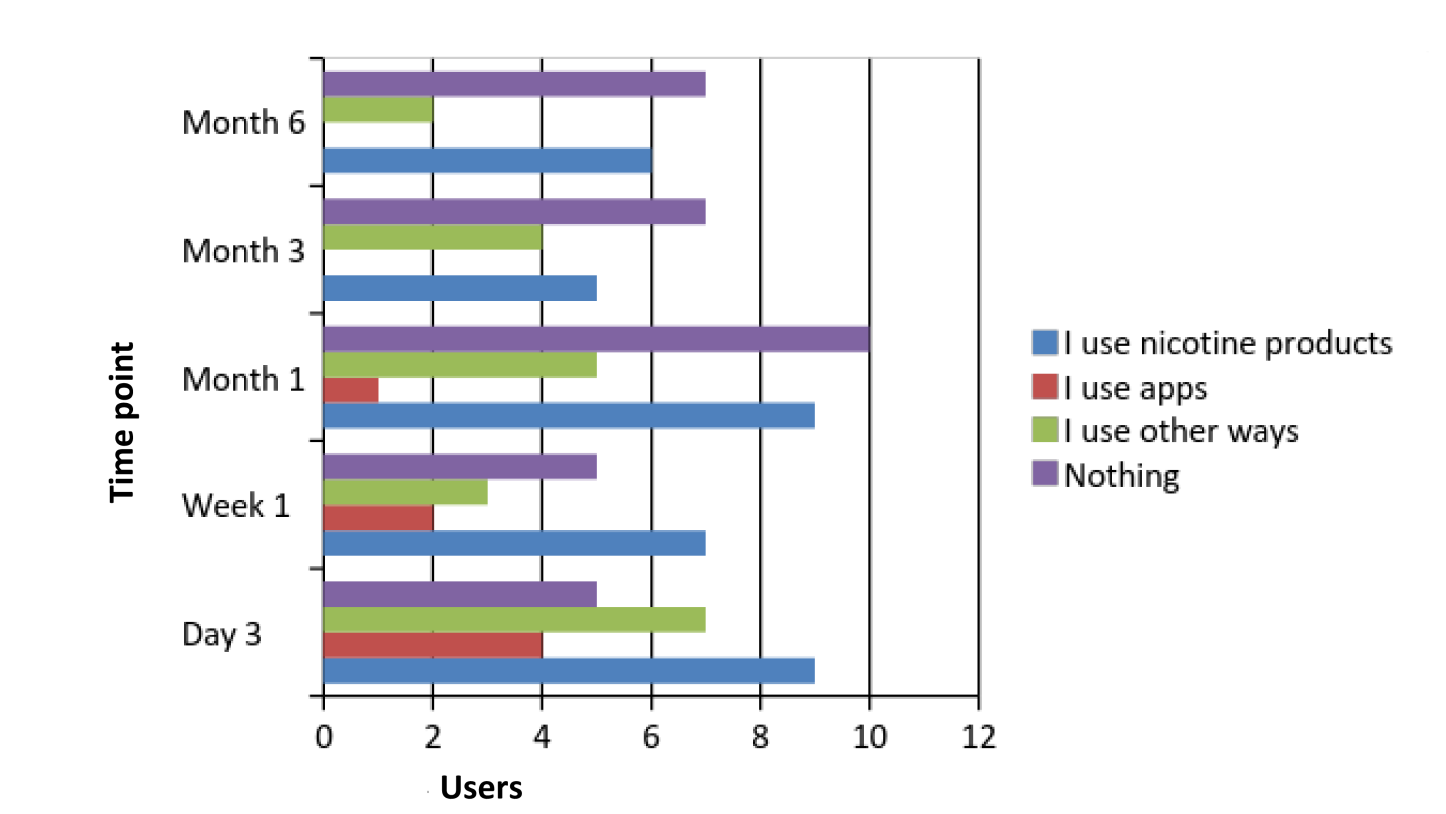
Fidelity responses of the comparison group detailing whether they used additional support.

Fidelity questions for the comparison group tried to identify whether participants used any kind of additional support to manage their cravings during the follow-up period. Figure 4 illustrates their responses to the closed-ended questions.

Participants’ responses to the open-ended question asking about what other methods they used when they faced cravings were as follows: pharmacological support (eg, Champix, nicotine replacement products); snus (smokeless tobacco); exercise, jogging, or running; walks in nature; and doing housework.

**Figure 5:**
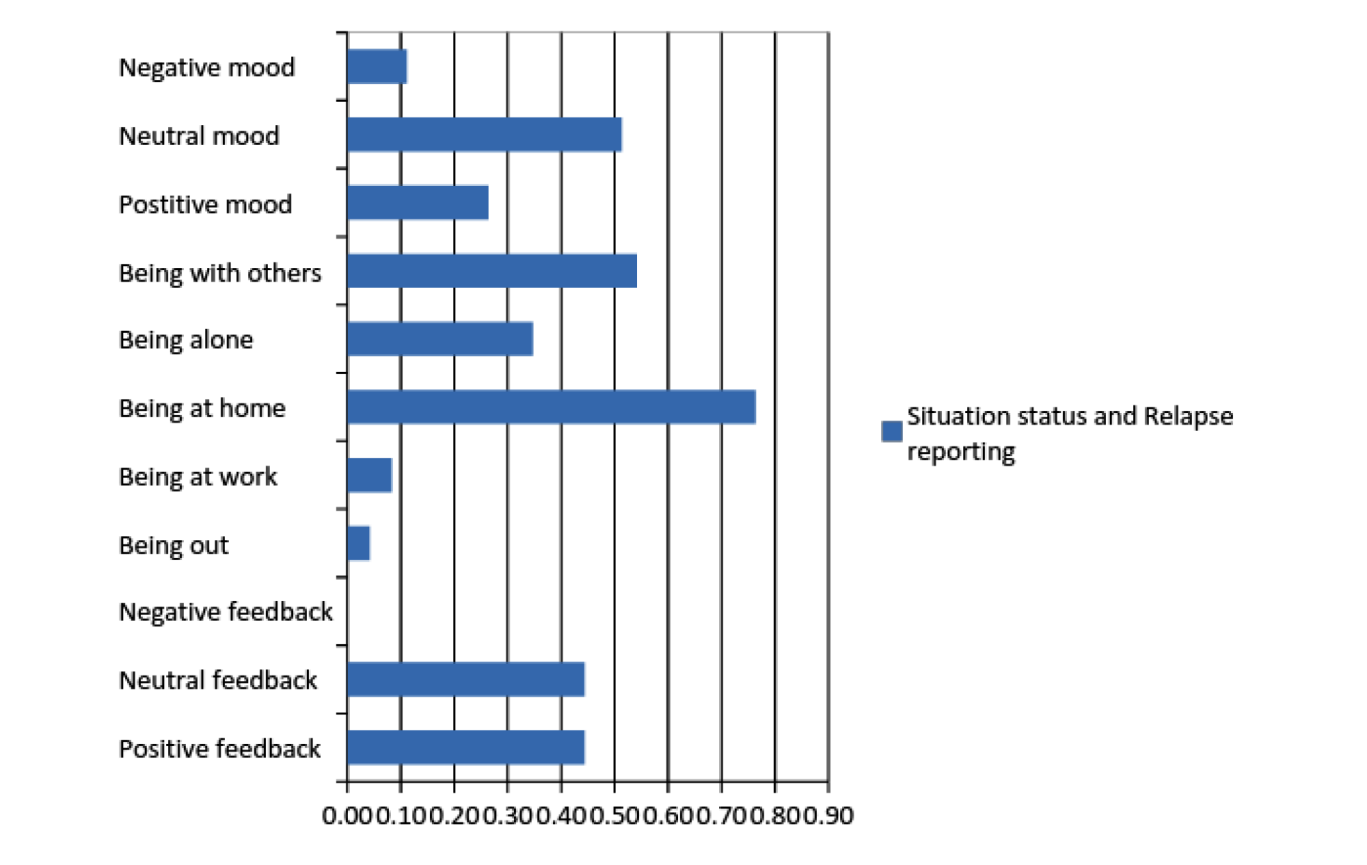
Average frequency of situation status and relapse reporting of all app users during the 6-month follow-up, based on phone data.

Table 4 displays phone data regarding the average frequencies of participants’ status (mood, place, social environment) and relapse reports at every measurement time point. Figure 5 illustrates the overall average frequency of situation status and relapse reporting of all app users, based on phone data, during the 6-month follow-up.

#### Usability

We assessed perceived quality of the PhoS app twice, at weeks 1 and 4, after participants had started using the app. Participants’ (n=23) average SUS score 1 week after they started using the app was 21.6 out of 100. Participants’ (n=20) scores were 21.8 at 1-month follow-up. This indicates a relatively low level of usability, considering that SUS scores below 68 are assumed to be below average [30]. Participants’ reasons for stopping using the app were that (1) they were trying to avoid using their phone outside of work time, (2) the app did not work on their phone and carrying a second phone for the express purpose of using the app was inconvenient, (3) some perceived the suggested tasks to be “weird,” (4) they did not feel the need to use the app and they could manage cravings without it, (5) it was not powerful enough to help them overcome their cravings, and (6) they did not use apps in general.

**Table 4:** Average frequencies of situation status and relapse reporting of app users at every measurement time point during follow-up.

Status	Time point							
	3 days	1 week	2 weeks	3 weeks	4 weeks	8 weeks	12 weeks	24 weeks
Positive feedback^a^	0.89	0.44	0.22	0.11	0.00	0.22	1.11	0.56
Neutral feedback^b^	1.11	0.33	0.44	0.44	0.33	0.56	0.22	0.11
Negative feedback^c^	0.00	0.00	0.00	0.00	0.00	0.00	0.00	0.00
Outdoors	0.22	0.00	0.00	0.11	0.00	0.00	0.00	0.00
At work	0.44	0.11	0.00	0.00	0.11	0.00	0.00	0.00
At home	1.33	0.67	0.67	0.44	0.22	0.78	1.33	0.67
Alone	0.89	0.44	0.22	0.44	0.11	0.22	0.00	0.44
Not alone	1.11	0.33	0.44	0.11	0.22	0.56	1.33	0.22
Positive mood	0.67	0.22	0.11	0.00	0.00	0.22	0.33	0.56
Neutral mood	1.00	0.44	0.56	0.44	0.33	0.44	0.78	0.11
Negative mood	0.33	0.11	0.00	0.11	0.00	0.11	0.22	0.00

^a^“The app was helpful to manage craving.”

^b^“I managed not to relapse, but the app didn’t help.”

^c^“I relapsed.”

### Preliminary Efficacy Outcomes

Overall, intention-to-treat analyses revealed that 36% (n=16) of the 44 participants who entered the follow-up period as quitters remained abstinent after 6 months. The abstinence proportion increased to 53% for those who provided complete data at all time measurement points (n=30) up to 6 months.

A logistic regression analysis examined the effects of intervention group (intervention vs control) for differences in the 7-day PPA at the 6-month follow-up period. Odds of quitting were not statistically significantly different between the groups for the intention-to-treat (n=44) and complete-case analyses (n=30). Multimedia Appendix 4 displays the frequencies and percentages of successful quitters during the 6-month follow-up period by group for the intention-to-treat and complete-case analyses.

We performed the same logistic regression analysis including use of pharmacological support at the end of the follow-up period as a covariate, since pharmacological support is strongly related to the main outcome. Table 5 presents descriptive measures. Intention-to-treat analysis revealed that the odds of quitting in the comparison group were lower for those who did not use any pharmacological support (odds ratio [OR] 0.23, 95% CI 0.02-2.59, *P*=.24), while odds of quitting in the intervention group were higher for those who did not use pharmacological support (OR 16.07, 95% CI 0.83-313). The group × pharmacological support interaction effect fell just short of statistical significance (χ^2^_40_=53.7, *P=*.05).

**Table 5:** Frequencies and percentages of successful quitters at the 6-month follow-up time point by group and use of pharmacological support (nonabstinent/abstinent) for intention-to-treat and complete-case analyses.

Study group		Intention-to-treat		Complete case	
		Nonabstinent	Abstinent	Nonabstinent	Abstinent
**Intervention group, n (%)**					
	Nonabstinent	12 (75)	4 (44)	5 (56)	1 (17)
	Abstinent	4 (25)	5 (56)	4 (44)	5 (83%)
	Total	16 (64)	9 (36)	9 (60)	6 (40)
Total (nonabstinent + abstinent)		25 (100)		15 (100)	
**Comparison group, n (%)**					
	Nonabstinent	7 (54)	5 (83)	3 (33)	5 (83)
	Abstinent	6 (46)	1 (17)	6 (67)	1 (17)
	Total	13 (68)	6 (32)	9 (60)	6 (40)
Total (nonabstinent + abstinent)		19 (100)		15 (100)	

The interaction effect was statistically significant in the complete-case analysis (χ^2^_26_=34.6, *P=*.01), indicating that, in the intervention group, the odds of quitting were higher for those who did not use pharmacological support (OR 62.8, 95% CI 1.73-2259, *P*=.02). In the comparison group, the odds of quitting were lower for those who did not use any pharmacological support (OR 0.10, 95% CI 0.0-11.29, *P*=.08). A 2 (intervention: intervention vs comparison) × 7 (time points: follow-ups at 1, 2, 3, 4, 8, 12, and 24 weeks postintervention) GLMM examined the effects of PhoS app use versus nonapp use for differences in 7-day PPA during the 6-month follow-up period. There was no statistically significant interaction between time trend and intervention group (χ^2^_1_=3.6, *P*=.06) for the intention-to-treat group. There was a statistically significant interaction effect between intervention group and use of pharmacological support (χ^2^_1_=4.1, *P*=.04), indicating that those participants in the intervention group who did not use pharmacological support were more likely to be abstinent during the entire follow-up period.

### Secondary Outcomes

Mann-Whitney *U* tests indicated that there were no statistically significant differences between groups for all secondary outcome measures (relapses, cravings, AEF, MCEF, and CCM) at the end of the follow-up period (6 months). Similarly, GLMMs indicated that there were no statistically significant differences between groups for the same secondary outcome measures during the entire follow-up period. Multimedia Appendix 5 displays the descriptive statistics of all secondary measures at all follow-up time points by group.

We included all secondary measures as covariates in the analysis examining the effect of the intervention and time point on the primary efficacy measure, abstinence proportion. Only AEF at the end of the 6-month follow-up period and the intervention group had a significant interaction effect (χ^2^_1_=4.2, *P*=.04) on abstinence for the complete-case analysis. More specifically, in the intervention group AEF increased abstinence levels, meaning that for every unit increase in AEF the odds of quitting were multiplied by 2.5. However, in the comparison group AEF decreased abstinence, meaning that for every unit increase in AEF the odds of quitting were multiplied by 0.77.

### Additional Outcomes

#### Physical Activity Behavior

Mann-Whitney *U* test for differences in IPAQ scores between session 3 and at the end of the 6-month follow-up period revealed significant differences (*U*=23, *P*=.008) between the intervention and comparison groups. This result indicates that the distribution of the difference in IPAQ scores between those time points was significantly different across groups. Intervention group participants reported higher physical activity scores (mean rank=16.20, n=10) than comparison group members (mean rank=8.77, n=13). The same analysis for testing the distribution of the difference in IPAQ scores between session 1 and the 6-month follow-up period revealed no statistically significant differences across groups (*U*=77, *P*=.55).

#### Theory of Planned Behavior Constructs of Physical Activity Behavior

We tested the role of the sociocognitive variables from the theory of planned behavior for physical activity in predicting secondary outcomes (number of cigarettes smoked, AEF, CCM, and MCEF) using linear multiple regression. Specifically, the dependent variable of interest was regressed on the theory of planned behavior variables controlling for treatment group, pharmacological support use, and time by using intention-to-treat and complete-case analyses. We conducted separate analyses for each dependent variable for complete cases. The number of cigarettes smoked was a dependent variable at all follow-up time points. Perceived behavioral control over physical activity was a statistically significant predictor of number of cigarettes smoked (B=–5.27, SE 2.18, *P*=.01) and MCEF (B=1.89, SE 0.57, *P*=.003). The negative coefficient indicated that a higher perceived behavioral control over physical activity score was related to fewer cigarettes smoked during all follow-up periods. An identical analysis using the theory of planned behavior measures taken at session 1 revealed no statistically significant results. There were no other statistically significant effects. The linear mixed-effects model, for complete cases, of MCEF × time was significant, indicating that as time was progressing, MCEF was decreasing (B=–0.75, SE 0.30, *P*=.02).

## Discussion

### Implementation Outcomes

Use of the PhoS app was lower than expected. Retention rates for app use declined throughout the follow-up period. The increase of retention rate at 6 months (30%) after the lower rate at 3 months (10%) implies that there was a group of stable users throughout the period. After locating those 4 participants, we determined that their probability of quitting compared with the comparison group was higher. Characteristics common to these participants were their annual income, having a higher education degree, not using any pharmacological support during the study, and considerably increasing their physical activity from baseline to the end of the 6-month follow-up period.

Intervention group participants reported that they used the app mostly when they were at home, with others, and in a neutral mood. Relapse reporting indicated that when they used the app they were able to successfully manage their cravings and not relapse. Nevertheless, the frequency of positive and neutral feedback indicates that the app was helpful for some participants but not others for relapse prevention. None of the phone entries reported a relapse. Qualitative data from the fidelity check questions revealed when, how, and why participants used, or did not use, the app. Most participants who reported using the app used it to plan what to do either before experiencing a craving or when they had actually experienced a craving. However, some reported that they used the app for other reasons also (for fun or just browsing).

### Usability

Usability level results were very low. The low SUS scores indicate that there is a need to review the app and identify usability problems. However, SUS itself is not diagnostic, so the results do not shed light on the reasons for low reported usability. One of the reasons was that, although participants were given the option to replace their phones with study mobile phones if the app could not operate on their own device, for several reasons some participants did not accept that. Using a second phone for accessing the app introduced an additional potential bias of usability. The open question for usability revealed some reasons for the low scores. Most of them are common reasons that apply to most apps: the person did not use apps in general or there were compatibility issues with their device. Fidelity questions regarding the reasons for not using the app were informative for the attrition in app use. For example, 1 participant reported that “After the initial excitement I don’t think it’s for me,” suggesting that the novelty decreases, and sustained engagement with these kinds of apps is low and has been previously reported as a reason for attrition in the use of mHealth apps. Research has indicated that users’ initial interest in mHealth apps quickly fades as the novelty wears off [35]. Nevertheless, statements such as “I don’t need it anymore” were made by users who stopped smoking, used the app for some time (as long as they experienced cravings), and then stopped using it once their cravings faded because there was no reason to continue. Finally, a few statements indicated that some suggested physical activities were “weird” or were not powerful enough to help participants overcome their cravings. Overall, there is significant room for improvement in increasing the novelty and usability of the PhoS app to improve sustained engagement and use.

### Preliminary Efficacy Outcomes

We found that intervention participants provided with the PhoS app and training on how to use it did not report greater abstinence or differences in outcomes relating to craving management relative to the comparison group. The lack of differences between the groups suggests that the app did not provide added value in assisting quitting or managing cravings. This result may be attributable to the low frequency of use and level of usability of the app. A further possible explanation may be that we tested the PhoS app against a strong comparison group in which participants received the same counseling smoking cessation program that was promoting physical activity as a means to manage cigarette cravings. The lack of any additional added effect of the app might be attributed to the relative strength of the effect of the counseling intervention in the comparison group. Responses to fidelity questions by comparison group participants implied that they also used several forms of physical activity as a method to manage their cravings (eg, exercise, jogging, or running, walks in nature, or housework). It would be interesting in a future study of the app to use a usual-care comparison group to test whether the app alone can have an added effect on abstinence rates.

The overall abstinence for all participants (n=44), after 6 months, was 36% in intention-to-treat analyses and 53% in complete-case analyses. These long-term abstinence rates are considered satisfactory, but while generalized comparisons can be made with other smoking cessation programs, no direct comparison can be made given that this intervention included several unique components: behavioral counselling to quit smoking, physical activity promotion, and pharmacological support. A previous study [36] reported abstinence rates of 42% (for cognitive behavior therapy plus nicotine replacement therapy) and 36% (for physical activity plus nicotine replacement therapy) at 12-month follow-up. Overall, our results compare favorably with these abstinence rates and support the use of physical activity as a means to manage smoking cravings in smoking cessation programs [13-15].

Pharmacological support was also an important moderator of the effects of the intervention on abstinence. Odds of quitting were lower among participants in the comparison group who did not use any pharmacological support, while odds of quitting were higher in the intervention group for those who did not use pharmacological support. These interactive effects were evident during the entire follow-up period. This finding implies that the app may be an effective supportive tool to promote abstinence instead of using pharmacological support. Although participants in the intervention group were free to choose to use pharmacological support if they wanted to, they were not asked why they did not use pharmacological support. Therefore, it is not clear whether this finding is caused by chance, since it was not possible to locate a similar finding in previous research, so this finding should be treated as preliminary. An interesting avenue for future research would be to examine whether mHealth apps can be used as an alternative means of support quitting smoking for people who cannot use, or do not want to use, pharmacological support.

### Secondary Outcomes

After testing all 5 secondary measures as covariates to the primary efficacy measure, there was a significant interaction between AEF and group, indicating that increased AEF and being a member of the intervention group increased the odds to stay abstinent. According to self-efficacy theory [37,38], developing awareness of specific situations where efficacy may be low and mentally rehearsing desired behavior in these situations appears to enhance efficacy for behavior change. This finding paves the way for future interventions promoting quitting smoking to identify situations where participants might lapse and visualize potential solutions or responses to direct them away from the typical response of lighting up a cigarette.

### Additional Outcomes

Although there were no differences between the groups of users and nonusers of the app in the main outcome, there were important group differences in some of the additional outcome measures. The differences between groups on self-reported physical activity behavior suggest that the app acted as a reminder of being more physically active as well. The question arising from this finding is whether any physical activity promotion app would have the same effect. According to a meta-analysis [39], mobile devices are an effective means for influencing physical activity behavior, but no study has tested the effect of these apps on smoking cessation.

In addition, higher perceived behavioral control over physical activity behavior, measured at session 4, was related to participants smoking fewer cigarettes and higher MCEF capacity at all follow-up periods, regardless of group allocation and pharmacological support. Since the same result did not apply to perceived behavioral control over physical activity behavior at session 1, it is likely that such changes were attributable to the smoking cessation intervention. Sociocognitive theories postulate that sense of control is the most powerful source of self-efficacy [22,40]. The possible mechanism behind this might be that successful attempts to control physical activity behavior had the effect of strengthening self-efficacy to manage cigarette cravings. Theorists support the existence of this mechanism, but they claim that this is possible mainly for the same behavior or domain [22,41], whereas, based on our data, this mechanism worked for another behavior. This is consistent with research demonstrating that sociocognitive beliefs translate across health behavior contexts [42]. Nevertheless, the focus of this study and the low number of participants precluded a test for a causal relationship and should be a priority for future research.

### Strengths and Limitations

To our knowledge, this is the first study to develop and test the feasibility, acceptability, usability, and preliminary efficacy of an mHealth mobile phone app that promotes physical activity as a means to manage smoking cravings. Lessons learned from this study may inform future research in this area. The small number of participants was an a priori limitation of this pilot study identified in the study protocol [19]. In addition, as is common to much research on smoking cessation and mHealth apps, retention was one of the main challenges faced in this study [35,43,44], and this had a direct effect on the power of the study results [45,46]. Moreover, generalizability and applicability are limited due to the low use and usability of the app.

### Future Research

We developed the PhoS app for research purposes, so the focus was on developing an app with a strong basis in theory- and evidence-based behavior change techniques. An equal focus on design, attractiveness, and usability would increase use. Therefore, future attempts to use the PhoS app as a tool should first focus on improving its usability. For that reason, the app code is available, upon request from the first author, for research purposes only.

Introducing the PhoS app earlier to the participants, during the smoking cessation intervention, when participants decrease the number of cigarettes and increase their physical activity, might improve use of the app (eg, more consistent and coherent use, at the right time and for the right reasons). This way there would be more time to practice using the app and provide opportunities to receive feedback on its use from those delivering the intervention.

Testing the PhoS app among people who have quit smoking with a method or program that does not use physical activity as a supportive aid might reveal useful information in a future trial. For example, an important question is whether the app can stand alone and help quitters manage their cravings through short bouts of physical activity when it is not used in conjunction with another intervention, such as the counselling quit-smoking intervention in this study. Another direction for future research should be to test the use of a physical activity promotion app as a supportive relapse prevention tool in quit-smoking interventions.

### Conclusion

Overall, implementation results from this study indicated that the PhoS app needs improvements before being embedded in a larger trial. Moreover, the app did not have an additive effect on abstinence based on smoking abstinence beyond the effects of the counselling quit-smoking intervention. Nevertheless, findings on the secondary dependent variables shed some light on the possible mechanisms that were activated by its use, independently and in combination with the counselling and pharmacological smoking cessation intervention. Despite the challenges of research on mHealth apps, there is documented potential and research on developing apps that are optimally engaging and usable, as well as an evidence from behavioral science, that these apps may assist in this potential being realized in the domain of smoking cessation.

## Appendix

Multimedia Appendix 1 CONSORT-EHEALTH checklist V1.6.1.

Multimedia Appendix 2 Physical activity over Smoking (PhoS) app screenshots.

Multimedia Appendix 3 Measures timeline.

Multimedia Appendix 4 Successful quitters.

Multimedia Appendix 5 Secondary outcomes descriptive statistics.

## Abbreviations

AEF: self-efficacy of being aware of experiencing cravings
CCM: power of control in managing cravings
GLMM: generalized linear mixed model
IPAQ: International Physical Activity Questionnaire
MCEF: self-efficacy in managing cravings
OR: odds ratio
PhoS: Physical activity over Smoking
PPA: point prevalence abstinence
SUS: System Usability Scale
