# Supplementary material for: An mHealth App for Supporting Quitters to Manage Cigarette Cravings With Short Bouts of Physical Activity: A Randomized Pilot Feasibility and Acceptability Study
Source: JMIR Mhealth Uhealth. 2017 May 26;5(5):e74. doi: 10.2196/mhealth.6252 (PMC5466703; doi:10.2196/mhealth.6252)
Supplement: Multimedia Appendix 3 [file mhealth_v5i5e74_app3.pdf]

	Time points																			
	**$-t_3$**	**$-t_2$**	**$-t_1$**	**t_0**	**t_1**		**t_2** (3d. after)		**t_3** (1w. after)		**t_4** (2w. after)		**t_5** (3w. after)		**t_6** (4w. after)		**t_7** (12w. after)		**t_8** (24w. after)	
	All	All	All	All	Com	Exp	Com	Exp	Com	Exp	Com	Exp	Com	Exp	Com	Exp	Com	Exp	Com	Exp
**Screening for eligibility**																				
**Intervention to quit Smoking (Session 1 & 3)**																				
**Quit day: Allocation**																				
**Intervention to manage cravings (4th session)**																				
**Follow-up**																				
**Measures**																				
Screening for Drug Use	x																			
Tobacco Dependence Screener	x																			
Motivation to Stop Smoking Scale	x																			
General Health Questionnaire	x																			
Physical Activity Readiness Questionnaire	x																			
Smoking behavior 7days		x	x		x	x														
7-day PPA							x	x	x	x	x	x	x	x	x	x	x	x	x	x
International Physical Activity Questionnaire		x	x																x	x
Power of control on manage cravings		x	x				x	x							x	x	x	x	x	x
Efficacy on being aware of experience cravings		x			x	x	x	x	x	x	x	x	x	x	x	x	x	x	x	x
Efficacy on managing cravings		x			x	x	x	x	x	x	x	x	x	x	x	x	x	x	x	x
Number of relapses last 7days					x	x	x	x	x	x	x	x	x	x	x	x	x	x	x	x
Number of cravings last 7days					x	x	x	x	x	x	x	x	x	x	x	x	x	x	x	x
TPB physical activity (attitude, PBC & intention)		x			x	x														
Cotinine in saliva					x	x														x
Fidelity check (Comparison group)							x		x						x		x		x	
Fidelity check (Experimental group)								x		x						x		x		x
Data from Ph.o.S app collection mechanism								x		x		x		x		x		x		x
Ph.o.S app Usability										x						x				

All = All participants; Com = Comparison group; Exp = Experimental group; PBC =Perceived behavioral control
