# Supplementary material for: An mHealth App for Supporting Quitters to Manage Cigarette Cravings With Short Bouts of Physical Activity: A Randomized Pilot Feasibility and Acceptability Study
Source: JMIR Mhealth Uhealth. 2017 May 26;5(5):e74. doi: 10.2196/mhealth.6252 (PMC5466703; doi:10.2196/mhealth.6252)
Supplement: Multimedia Appendix 4 [file mhealth_v5i5e74_app4.pdf]

| Time | Group | Intention to treat | | | Complete case | | |
|---|---|---|---|---|---|---|---|
| | | Non-abstinent N (%) | Abstinent N (%) | Total N (%) | Non-abstinent N (%) | Abstinent N (%) | Total N (%) |
| 1 week | Experimental | 6 (24%) | 19 (76%) | 25 (100%) | 6 (24%) | 19 (76%) | 25 (100%) |
| | Comparison | 9 (47.4%) | 10 (52.6%) | 19 (100%) | 9 (47.4%) | 10 (52.6%) | 19 (100%) |
| | Total | 15 (34.1%) | 29 (65.9%) | 44 (100%) | 15 (34.1%) | 29 (65.9%) | 44 (100%) |
| 2 weeks | Experimental | 11 44.0% | 14 56.0% | 25 100.0% | 7 33.3% | 14 66.7% | 21 (100.0%) |
| | Comparison | 9 47.4% | 10 52.6% | 19 100.0% | 8 44.4% | 10 55.6% | 18 100.0% |
| | Total | 20 45.5% | 24 54.5% | 44 100.0% | 15 38.5% | 24 61.5% | 39 100.0% |
| 3 weeks | Experimental | 8 32.0% | 17 68.0% | 25 100.0% | 6 26.1% | 17 73.9% | 23 100.0% |
| | Comparison | 6 31.6% | 13 68.4% | 19 100.0% | 5 27.8% | 13 72.2% | 18 100.0% |
| | Total | 14 31.8% | 30 68.2% | 44 100.0% | 11 26.8% | 30 73.2% | 41 100.0% |
| 4 weeks | Experimental | 9 36.0% | 16 64.0% | 25 100.0% | 6 27.3% | 16 72.7% | 22 100.0% |
| | Comparison | 6 31.6% | 13 68.4% | 19 100.0% | 6 31.6% | 13 68.4% | 19 100.0% |
| | Total | 15 34.1% | 29 65.9% | 44 100.0% | 12 29.3% | 29 70.7% | 41 100.0% |
| 8 weeks | Experimental | 13 52.0% | 12 48.0% | 25 100.0% | 7 36.8% | 12 63.2% | 19 100.0% |
| | Comparison | 7 36.8% | 12 63.2% | 19 100.0% | 4 25.0% | 12 75.0% | 16 100.0% |
| | Total | 20 | 24 | 44 | 11 | 24 | 35 |

| | | | | | | | |
|---|---|---|---|---|---|---|---|
| | | 45.5% | 54.5% | 100.0% | 31.4% | 68.6% | 100.0% |
| 12 weeks | Experimental | 19 | 6 | 25 | 11 | 6 | 17 |
| | | 76.0% | 24.0% | 100.0% | 64.7% | 35.3% | 100.0% |
| | Comparison | 10 | 9 | 19 | 6 | 9 | 15 |
| | | 52.6% | 47.4% | 100.0% | 40.0% | 60.0% | 100.0% |
| | Total | 29 | 15 | 44 | 17 | 15 | 32 |
| | | 65.9% | 34.1% | 100.0% | 53.1% | 46.9% | 100.0% |
| 24 weeks | Experimental | 16 | 9 | 25 | 6 | 9 | 15 |
| | | 64.0% | 36.0% | 100.0% | 40.0% | 60.0% | 100.0% |
| | Comparison | 12 | 7 | 19 | 8 | 7 | 15 |
| | | 63.2% | 36.8% | 100.0% | 53.3% | 46.7% | 100.0% |
| | Total | 28 | 16 | 44 | 14 | 16 | 30 |
| | | 63.6% | 36.4% | 100.0% | 46.7% | 53.3% | 100.0% |
