# Supplementary material for: An mHealth App for Supporting Quitters to Manage Cigarette Cravings With Short Bouts of Physical Activity: A Randomized Pilot Feasibility and Acceptability Study
Source: JMIR Mhealth Uhealth. 2017 May 26;5(5):e74. doi: 10.2196/mhealth.6252 (PMC5466703; doi:10.2196/mhealth.6252)
Supplement: Multimedia Appendix 5 [file mhealth_v5i5e74_app5.pdf]

Follow up time points	Group	Mean (SD)				
		Relapses	Cravings	AEF	MCEF	CCM
1 week	Comparison	.48 (1.00)	34.15 (60.32)	9.21 (.91)	8.05 (1.84)	4.19 (1.39)
	Experimental	.68 (.88)	21.21 (28.03)	8.92 (1.35)	8.48 (1.98)	4.51 (1.30)
2 weeks	Comparison	3.38 (13.01)	12.11 (14.98)	9.00 (1.08)	8.50 (1.82)	-
	Experimental	.67 (.90)	15.19 (28.98)	9.25 (1.11)	8.20 (2.04)	-
3 weeks	Comparison	.52 (.99)	10.55 (12.47)	8.79 (1.31)	8.74 (1.40)	-
	Experimental	.58 (.96)	12.61 (23.82)	9.04 (1.33)	8.43 (2.40)	-
4 weeks	Comparison	.82 (1.46)	2133 (48.59)	9.11 (1.24)	8.42 (1.64)	4.63 (1.39)
	Experimental	.84 (1.64)	9.90 (15.23)	8.95 (1.78)	8.45 (2.19)	4.32 (1.57)
8 weeks	Comparison	2.42 (5.91)	16.53 (25.19)	9.25 (1.00)	8.06 (2.59)	-
	Experimental	4.37 (15.67)	8.72 (21.28)	8.68 (2.13)	8.05 (3.20)	-
12 weeks	Comparison	14.40 (36.97)	15.40 (29.11)	9.06 (1.27)	7.93 (2.89)	4.73 (1.76)
	Experimental	4.60 (8.16)	27.05 (38.97)	9.00 (1.77)	6.80 (3.23)	3.98 (2.06)
24 weeks	Comparison	3.47 (5.68)	24.33 (37.50)	9.07 (1.87)	6.07 (4.14)	4.87 (2.07)
	Experimental	9.71 (22.25)	13.36 (24.70)	7.80 (3.05)	7.07 (3.53)	4.03 (2.17)

Note: AEF = Awareness Efficacy; MCEF = Manage Cravings Efficacy; CCM = Cravings Control Management. There were no significant between group differences detected in the means
